# Speech-Like Rhythm in a Voiced and Voiceless Orangutan Call

**DOI:** 10.1371/journal.pone.0116136

**Published:** 2015-01-08

**Authors:** Adriano R. Lameira, Madeleine E. Hardus, Adrian M. Bartlett, Robert W. Shumaker, Serge A. Wich, Steph B. J. Menken

**Affiliations:** 1 Institute for Biodiversity and Ecosystem Dynamics, University of Amsterdam, Amsterdam, the Netherlands; 2 Pongo Foundation, Oudewater, the Netherlands; 3 Neuroscience Institute, Department of Psychology, Princeton University, Princeton, New Jersey, United States of America; 4 Indianapolis Zoo, Indianapolis, Indiana, United States of America; 5 Anthropology Department, Indiana University, Bloomington, Indiana, United States of America; 6 Research Centre in Evolutionary Anthropology and Palaeoecology, School of Natural Sciences and Psychology, Liverpool John Moores University, Liverpool, United Kingdom; Universite Paris XI - CNRS, France

## Abstract

The evolutionary origins of speech remain obscure. Recently, it was proposed that speech derived from monkey facial signals which exhibit a speech-like rhythm of ∼5 open-close lip cycles per second. In monkeys, these signals may also be vocalized, offering a plausible evolutionary stepping stone towards speech. Three essential predictions remain, however, to be tested to assess this hypothesis' validity; (i) Great apes, our closest relatives, should likewise produce 5Hz-rhythm signals, (ii) speech-like rhythm should involve calls articulatorily similar to consonants and vowels given that speech rhythm is the direct product of stringing together these two basic elements, and (iii) speech-like rhythm should be experience-based. Via cinematic analyses we demonstrate that an ex-entertainment orangutan produces two calls at a speech-like rhythm, coined “clicks” and “faux-speech.” Like voiceless consonants, clicks required no vocal fold action, but did involve independent manoeuvring over lips and tongue. In parallel to vowels, faux-speech showed harmonic and formant modulations, implying vocal fold and supralaryngeal action. This rhythm was several times faster than orangutan chewing rates, as observed in monkeys and humans. Critically, this rhythm was seven-fold faster, and contextually distinct, than any other known rhythmic calls described to date in the largest database of the orangutan repertoire ever assembled. The first two predictions advanced by this study are validated and, based on parsimony and exclusion of potential alternative explanations, initial support is given to the third prediction. Irrespectively of the putative origins of these calls and underlying mechanisms, our findings demonstrate irrevocably that great apes are not respiratorily, articulatorilly, or neurologically constrained for the production of consonant- and vowel-like calls at speech rhythm. Orangutan clicks and faux-speech confirm the importance of rhythmic speech antecedents within the primate lineage, and highlight potential articulatory homologies between great ape calls and human consonants and vowels.

## Introduction

Speech is a human hallmark, but its evolution remains enigmatic. Speech is organized in series of open-close mouth cycles where the opened phase essentially corresponds to vowel production and the closed phase to consonant production [1]. Characteristically, these cycles occur at 3–8 times per second (i.e. Hz) [2], the motoric outcome of rapidly stringing together consonants and vowels to produce the syllables, words and sentences that comprise the world's spoken languages. While nonhuman primates, including great apes, are known to combine calls sequentially (e.g. [3], [4]) and to flexibility combined them to produce meaningful differences (e.g. [5]), only recently a primate signal exhibiting a speech-like rhythm has been identified [2] – monkey (*Macaca* sp.) facial signals, known as lip smacks. Lip smacks show an idiosyncratic ontogeny [2] and differ coordinatively from chewing [2]. Moreover, monkeys are perceptually tuned to lip smacks at speech-like rhythm other than slower or quicker versions [2]. Despite being visual signals, lip smacks in monkeys suggest that speech rhythm may have evolved from ancestral mute signals and therefore lip smacks have been proposed putative speech precursors [2]. Initial support to this hypothesis has been lent by similar species-specific lip smacks in gelada baboons (*Theropithecus gelada*) which are vocalized [6]. Speech-like rhythm in monkeys may not be restricted, thus, to the visual modality, but also involve the acoustic domain.

Despite these recent results, and their potential relevance for our understanding of speech evolution, this hypothesis remains evolutionarily “jammed” because these monkey behaviours show to date no parallel in great apes – our closest relatives. This stance creates a time gap of approximately 25mya between speech-rhythm in old-world monkeys and humans [7], raising questions whether the former could have represented the precursor of the latter. To confirm conclusively an evolutionary link between the two, and attest this hypothesis' validity, three critical predictions remain to be assessed. First, great apes should also produce signals at a speech-like rhythm. Second, speech-rhythm should involve both vowel- as well as consonant-like calls, as speech rhythm is the direct manifestation of assembling together these two basic speech building blocks. Third, as in humans, speech-rhythm should be experience-based or socially learned, instead of being inherited, as observed in monkeys (monkeys lip smacking [8], geladas wobble vocalizations [9]). The main aim of the current paper is to analyse two newly described call types produced by a captive orangutan (*Pongo pygmaeus*) female which address these three predictions.

## Material and Methods

### Subject

Tilda (Studbook ID: 1452) is a Bornean female orangutan (*Pongo pygmaeus*), currently housed at the Cologne Zoo, Germany. She was born in the wild on the island of Borneo approximately in 1965. The exact location of capture is unknown. She was captured when she was ∼2 years-old, but her first register dates from 1975, that is, 8 years after capture, when she became privately owned by Hugo Steiner in Studen, Swizterland. Between 1975 and 2007 she was housed at the Zoo Seeteufel (retained by Steiner family), after which she was transferred in 2007 to Zoo Krefeld, Germany, where she remained one year before being loaned to the Cologne Zoo.

The lack of any records for the large part of Tilda's infancy and early adolescence (i.e. 2 – 10 years-old) and her purchase by a private collector in 1975, suggest that she was maintained during these years in private European circles. Before 1975, it is probable that Tilda was trained for human entertainment, possibly Belgium, as suggested by her caretakers and patently shown in several of her human-like behaviours otherwise never seen in the wild, such as human whistling [10], hand-clapping and arm-waving. No historical records that could elucidate the origin and ontogeny of Tilda's atypical calls exist.

### Data collection and analyses

Video and audio recordings were collected at Cologne Zoo in April 2010 with a Sony HDV 1080i (Sony Corporation, Japan) and Marantz PMD660 (Marantz, Japan), at 1m away through enclosure mesh. All recorded calls were used for analyses, that is, there were no selection criteria for inclusion of data for analyses. Video was digitally recorded at 25 frames/sec, using 720×576px resolution and 8-bit color. Audio was recorded at 48kHz sampling frequency and 16-bit resolution. Recordings were collected by the authors during food provisioning by Tilda's caretaker (Mike Ebert).

Clicks and faux-speech produced by Tilda were deemed to be communicative signals because Tilda only produced these calls in the presence of and directly facing her caretakers during feeding time, and she assisted her calls several times with pointing with her protruded lip and/or index finger towards the food in the caretaker's hand. Informal report by Tilda's caretakers, who accompany her daily, indicated that clicks and faux-speech were solely produced towards caretakers at feeding time, hence, data was solely collected at this time to avoid inducing added stress on the subject. Similar conditions have been found in chimpanzees, where novel calls were similarly used to draw caretakers attention [11]. Our main aim was to document, describe and compare the rhythm of Tilda's clicks and faux-speech to speech rhythm, and so we limited our data collection accordingly. Determining the precise behavioural and motivational correlates of Tilda's calls will require a more intense and extensive data collection in the future. Data collection did not involve direct interaction of the authors with the subject and was carried out in strict accordance with the guidelines and recommendations indicated by the subject's caretaker present during data collection.

Videos were loaded into MATLAB R2009b (MathWorks; Natick, MA) using *mmread* function. Upper and lower bounds of the midpoint of both lips were manually marked per frame, visually interpolating from adjacent frames when these points were covert. Euclidean distances between lips across time were calculated using MATLAB custom scripts. Video amplitude envelope was obtained via Hilbert transform's absolute value of the stereo channel with the highest amplitude. Amplitude modulation spectrum (0–100 Hz) was calculated via multi-taper Fourier transform of the amplitude envelope time series. Hilbert and Fourier were calculated respectively using MATLAB and Chronux Signal Processing Toolbox [12].

Orthogonal Slepian tapers (TW = 3, K = 5) were used for spectral analyses. Each video was split into 2 sec segments without overlap. Trailing segments <2 sec were excluded. Each segment was subsequently fitted with a first-order polynomial (using *polyfit* and *polyval* in MATLAB), subtracted from the original segment (to remove linear drift or DC component), and treated as an independent sample. For coherency estimation, amplitude envelope was down-sampled to the video sampling rate prior to z-scoring and detrending. Power spectra confidence intervals were based on parametric Gaussian approximations, and coherence confidence intervals based on a leave-one-out jackknifing procedure [12]. Power spectra were fit for 1/f^α^ trends using linear regression on the logarithm of frequency and of power [13]. Formant and fundamental frequency calculations were made using Praat 5.3.64 [14] using standard settings for automated fundamental frequency extraction, except for voicing and silence thresholds, which were set to 0.25 and 0.2, respectively. We selected formant extraction for five formants based on visual inspection of the spectrograms.

For statistical comparison between click and faux-speech rhythmicity and that of other species-typical orangutan rhythmic calls, we used data on long-calls due to the relatively large sample size of recordings available for this call type. Long-calls were selected solely from Bornean individuals, that is, the same species as Tilda, in order to avoid inserting biasing factors into the comparison (for instance, long-calls have been described to exhibit substantial geographical variation [15], [16]). Namely, long-calls were selected from the Tuanan population (Mawas, Kalimantan Tengah, Indonesia), which exhibits some of the highest known numbers of flanged males within the same population [17], providing one of the widest natural range of variation in rhythmicity in orangutan long-calls, while controlling for biasing ecological and genetic factors. Statistical tests with lorks and fast long calls, two other known orangutan rhythmic calls, were not conducted because these calls are extremely rare (approximately 1 call per 2500 observation hours and 1 call per 800 observation hours, respectively) and sample size of available recordings was <2, hindering any suitable statistical comparison. For statistical comparison between click and faux-speech rhythmicity and orangutan chewing rates, we used chewing data on fruit because chewing rates on this food item are the fastest observed in orangutans, offering therefore the most stringent point of comparison with click and faux-speech rhythmicity, with chewing rates on other food items, such as leaves, showing lower values. Statistical tests were conducted using IBM SPSS 20 (2011, SPSS, Inc.), with significance level set at P<0.05.

## Results

**Figure 1:**
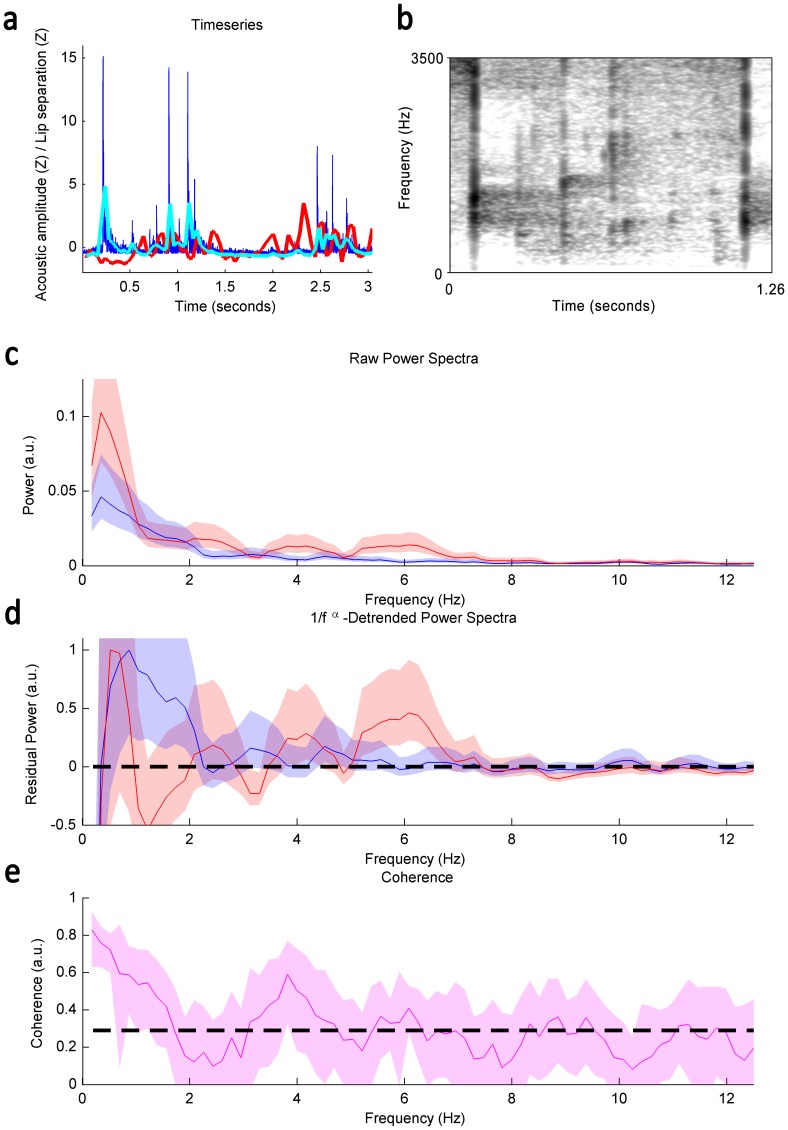
Descriptive analyses of orangutan clicks; a) Time series of an orangutan click bout. Dark blue line indicates raw power spectra (dB), light blue line indicates acoustic amplitude envelop measure, and red line indicates inter-lip distance measure. **b)** Spectrographic representation; **c)** Average estimates of raw power spectra; **d)** Detrended power spectra; **e)** Coherence of orangutan clicks.

**Figure 2:**
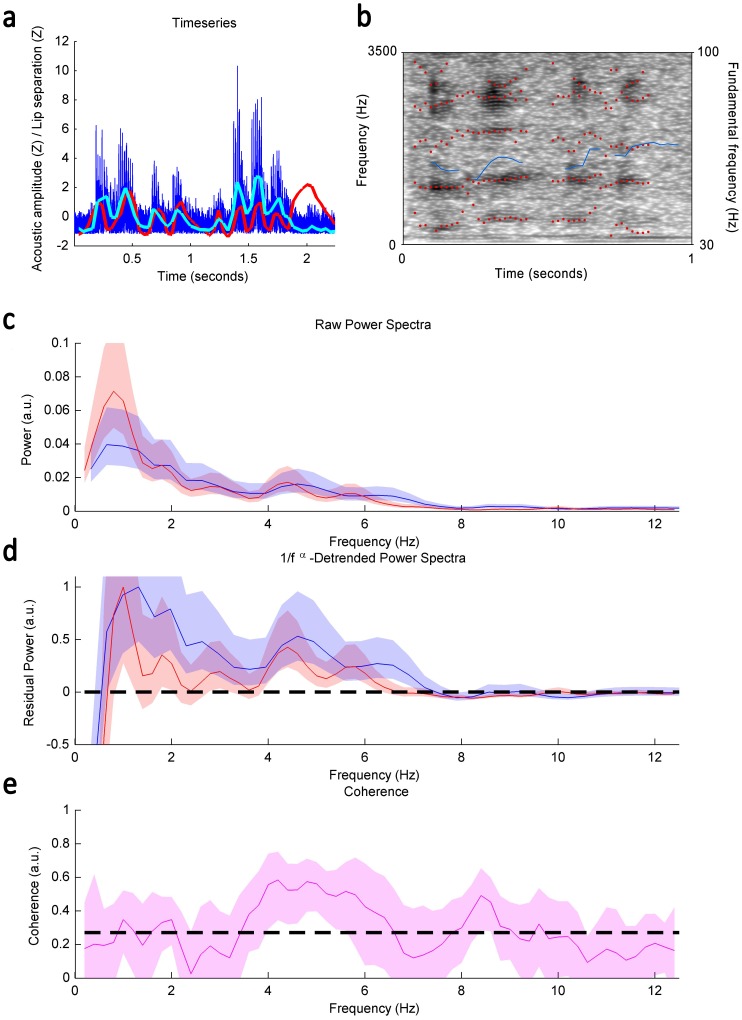
Descriptive analyses of orangutan faux-speech; a) Time series of an orangutan faux-speech bout. Dark blue line indicates raw power spectra (dB), light blue line indicates acoustic amplitude envelop measure, and red line indicates inter-lip distance measure. **b)** Spectrographic representation of faux speech. Red dots indicate formats and blue line indicates fundamental frequency measure. **c)** Average estimates of raw power spectra; **d)** Detrended power spectra; **e)** Coherence of orangutan faux-speech.

Tilda, an ex-entertainment orangutan housed at Cologne Zoo, Germany, in order to gather attention from her caretakers, produced a rhythmic voiceless call – “clicks” – and voiced call – “faux-speech”. Orangutans are known to produce both voiceless (e.g. kiss-sounds, raspberries) [18], [19] and voiced calls (e.g. long calls) [18], however, unlike any of these, clicks and faux-speech showed rapid lip movements (Figs. 1a, 2a, S1 Video, S2 Video, S1 Audio). To quantify the rhythm of these calls and their potential similarities with human speech rhythm, we analysed lip movements and amplitude envelopes using video and audio recordings [13] (Figs. 1a, 2a).

Clicks (*n*_bouts_ = 7, *n*_rhythmic lip cycles_ = 29) presented a mean bout duration (25; 75% percentiles) of 0.925 sec (0.74; 1.956) and a mean of 3 lip cycles per bout (2.5; 4). Via cinematic analyses, lip rhythmicity showed peaks at >1, 2, 4 and 6 Hz during click production, whereas acoustic amplitude rhythmicity presented peaks at >2, ∼3.5,∼4.5 Hz (Fig. 1c). Detrended power spectra showed that lip movements and acoustic amplitude were sometimes unsynchronized (Fig. 1d), indicating that sound production did not occur constantly at the lips, but resulted instead from tongue maneuvers. Nevertheless, significant high levels of coherence (i.e. phased synchronicity) between lip movements and acoustic amplitude were reached at >1.5 Hz and at speech-like rhythm of ∼4 Hz, where the lower bound of the 95% confidence interval of the coherence value (i.e. the lower bound of the shaded region) exceeded the theoretical criterion of 95% confidence interval (i.e. the dashed black line) (Fig. 1e). Significant coherence was also nearly reached at 6 Hz. Coherence levels at >1.5 Hz reflected the ∼1/f distribution of inter-bout intervals, a phenomenon known to emerge in analyses of animal and human utterances. Clicks showed energy bands across the frequency spectrum and extremely short durations (∼5 ms; Fig. 1b) typical of noisy bursts, as a hand clap. Clicks revealed neither formants nor narrow energy band(s) corresponding to harmonics (Fig. 1b).

Faux-speech (*n*_bouts_  =  8, *n*_rhythmic lip cycles_  =  43) presented a mean bout duration of 1.365 sec (1.009; 2.0) and a mean of 5 lip cycles per bout (4; 6.75). Faux-speech showed rhythmicity peaks for lip movement and acoustic amplitude between 4.5 and 6 Hz (Fig. 2c, d), indicating synchronous phasing between these two features, as confirmed by significant levels of coherence (Fig. 2e). Significant coherence was also nearly reached at ∼8.5 Hz. The absence of the >1.5 Hz component, as seen in clicks, is likely an artifact of faux-speech not displaying long periods (≥1s) of silence between bouts, as was the case for click recordings. Spectrographic measures showed that fundamental frequency varied between approximately 50 Hz and 95 Hz (Fig. 2b), indicating some exercise of the laryngeal musculature. During faux-speech formants also shifted (Fig. 2b), indicating positional changes of the supralaryngeal articulators, namely lips, tongue and/or jaw.

Using the largest database ever assembled of orangutan calls [18], currently including 7 wild and 6 captive populations, we found that orangutan calls consisting of rhythmic mouth movements did not surpass rhythms of 1 Hz, namely, long calls (∼0.5 Hz; [18], [20]), fast long calls (∼0.6 Hz; [18]) and lorks (∼0.4 Hz; [18]). These rhythms were significantly lower than those observed for clicks and faux-speech (Mann-Whitney U: *n*_long calls_ = 25, *n*_rhythmic lip cycles_ = 529; exact *P* = 0.000). Clicks and faux-speech rhythm was also faster than orangutan chewing rates as observed during feeding on fruit (∼1.3 Hz; Hardus, unpublished data), insects (∼1.3 Hz; [21]), leaves (∼1.2 Hz; [21]) and vertebrate meat (∼1.2 Hz; [21]), reaching significant levels (Mann-Whitney U: *n*_fruit chewing rates_ = 40, *n*_rhytmic lip cycles_ = 550; exact *P* = 0.000). Thus, orangutan clicks and faux-speech rhythm significantly surpassed by *at least* seven- and three-fold that of other similar communicative and non-communicative behaviours in orangutans.

## Discussion

Orangutan clicks and faux-speech verify that primate signals exhibiting a speech-like rhythm occur in a great ape. Rhythmic differences with chewing rates were in line with findings in monkeys [2] and humans [22]. Importantly, significant rhythmic differences were found in comparison with all other known rhythmic calls in orangutans. This comparison was based on the largest database ever assembled of the orangutan repertoire [18], currently including more than 6000 observation hours and more than 110 individuals of all sex-age classes. The probability that click and faux-speech rhythmicity was not compared with that of an unknown rhythmic orangutan species-specific call is, thus, highly unlikely. Moreover, besides clicks and faux-speech, no orangutan rhythmic call is known to be produced in affiliative contexts [18]. Rhythmic and contextual idiosyncrasies of clicks and faux-speech attest therefore that these calls do not simply correspond to variations of previously described calls [18].

Our findings differ however in a critical manner from previous observations in monkeys. Speech-like rhythm has been described in monkey signals characteristic of the species, whereas clicks and faux-speech are seemingly individual-specific. Tilda is an ex-entertainment orangutan and currently the only Bornean and wild-born orangutan capable of whistling, a voiceless call socially-acquired from humans or conspecifics [10]. It is tentative therefore to suggest that Tilda may have socially learned clicks and faux-speech through human training and practice during her long time in captivity.

Other alternatives include the possibility that these calls were invented *de novo* by Tilda, that they were ecologically facilitated, or that they were inherited. The first possibility is, however, unlikely, as this would mean that speech rhythm synchronous with call production would have spontaneously emerged twice in the same individual over the course of less than 30 years, while remaining undescribed in all other great ape individuals, ever since the first primate ethological studies started approximately one century ago [23], [24]. Possible ecological or genetic explanations seem also improbable. On the one hand, Tilda's physical environment in the wild and in captivity has been fundamentally similar to that of any other orangutan, with the exception of her close contact to humans since early age. On the other hand, no inherited call type with distinct acoustic and structural characteristics is specific solely to one individual. Even if Tilda came from an unstudied or extinct Bornean population where clicks and faux-speech were customary, these calls would still exhibit no counterparts in other extant populations, raising questions whether they would be rooted in genetics or ecology. Moreover, to our knowledge, no primate gene or mutation has been identified to solely affect acoustic oro-facial movements. Accordingly, the full scope of observations on Tilda indicate a possible role of social learning and training in the acquisition of voiceless and voiced calls in orangutans, aligning with observations made in the wild, where social learning has been inferred as the cause of geographic differences in call repertoire composition between orangutan populations, concerning both voiceless and voiced calls[25]–[28].

Specifically, our findings indicate for the first time that fine motor control in great apes may expand autonomously over tongue manoeuvring (see [10]). If tongue movements were otherwise tightly coupled with those of other vocal structures (such as the lips), lip and acoustic rhythmicity would be synchronous at all times, which was not the case during click production. In addition, orangutan faux-speech suggests that some degree of voluntary control may expand onto the vocal folds, at least over the lateral cricoarytenoid muscles responsible of the setting of the vocal folds in (adducted) position for phonation. Frequency range of faux-speech only overlaps with that of other three known orangutan calls; grumphs, gorkums and grumbles [18]. While the observed frequency modulations of faux-speech remained below 100 Hz, mean frequencies for these three other call types are found between 185 and 270 Hz, and their frequency range can exceed 1700 Hz [18]. Moreover, grumphs, gorkums and grumbles represent alarm and arousal calls [18]. It is therefore doubtful if Tilda's faux-speech “simply” derived from the tallying of speech-like lip oscillation to a known orangutan call. Even if this was the case, this would demonstrate that an articulatory innovation can give raise to new and distinct voiced call types. However, rhythmicity between clicks and faux-speech was not precisely the same, indicating that the emergence of these calls likely involved a more complex mechanism, and that some voluntary control over vocal fold activity may have been involved. Nevertheless, when setting aside putative origins and underlining mechanisms, clicks and faux-speech demonstrate that at least orangutans, but possibly also other great apes, show irrevocably no constrains at the respiratory, articulatory, and neurological level hindering the production of consonant- and vowel-like calls at speech-rhythm.

A brief terminological clarification is also required. Many animal species produce “clicks” (e.g. moths, swiflets, bats, whales, dolphins), and this is indeed a common word used colloquially to describe sounds in our daily lives. As demonstrated by our results, orangutan clicks differ altogether from other animal examples in that they result from the close articulatory manoeuvring of the lips and tongue. As such, orangutan resemble more human clicks, voiceless consonants that occur in approximately in 2% of the worlds' spoken languages and that result from the quick downward movement of the tongue apart from the palate [29]. The definition and description of other “clicks” across the animal kingdom is based on acoustic, other than articulatory, resemblance. Whether tongue and lip coordination between orangutan and human click production is exactly similar will require further analyses, such as via cineradiography.

In conclusion, orangutan clicks and faux-speech corroborate the importance of monkey rhythmic facial signals [2] and their vocalized counterparts [6], and demonstrate that besides human consonant and vowels, both voiced and voiceless calls at a speech-like rhythm may be produced by the same great ape individual. The extent of motoric control that great apes exert over their vocal structures, both laryngeal and supra-laryngeal, may be much higher than hitherto presumed, allowing the expansion of the call repertoire to include idiosyncratic voiced and voiceless calls. Moreover, great ape voiced and voiceless calls may provide useful models of human vowel and consonant antecedents [19]. A renewed interest in great ape call repertoires, on how consonant- and vowel-like calls are used in the wild and captivity, and how these two call categories function, will likely bring us closer to understand the conditions that brought together for the first time the two basic building blocks of speech.

## Supporting Information

S1 Audio **Compilation of faux-speech produced by Tilda.**
(WAV)

S1 Video **Faux-speech produced by Tilda.**
(WMV)

S2 Video **Clicks produced by Tilda.**
(WMV)
